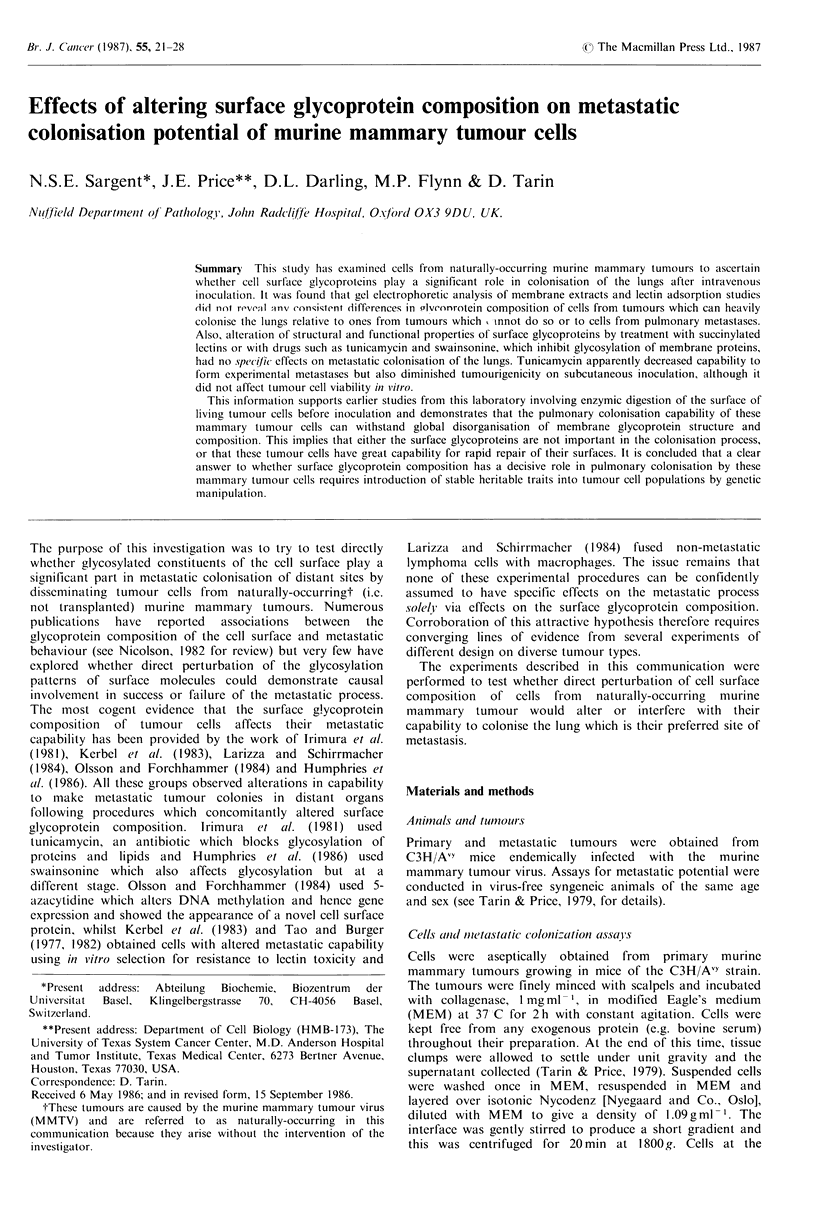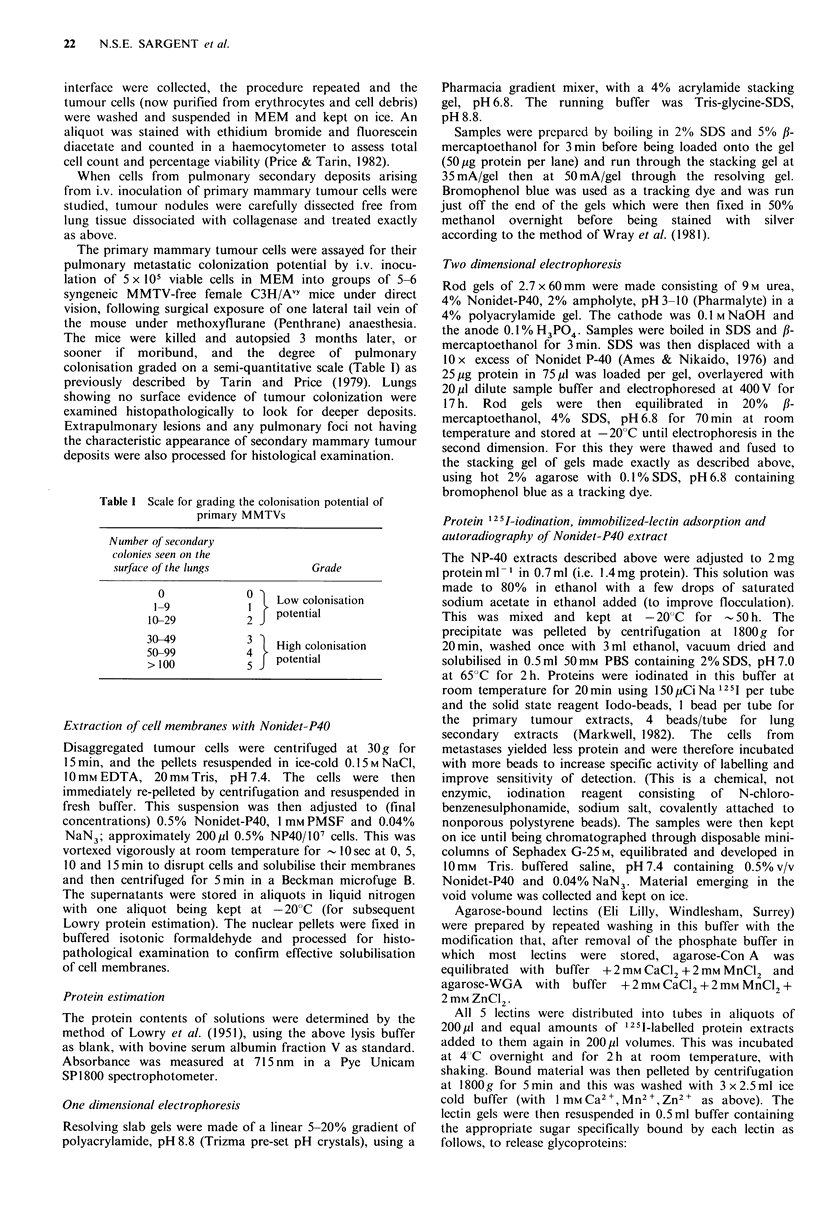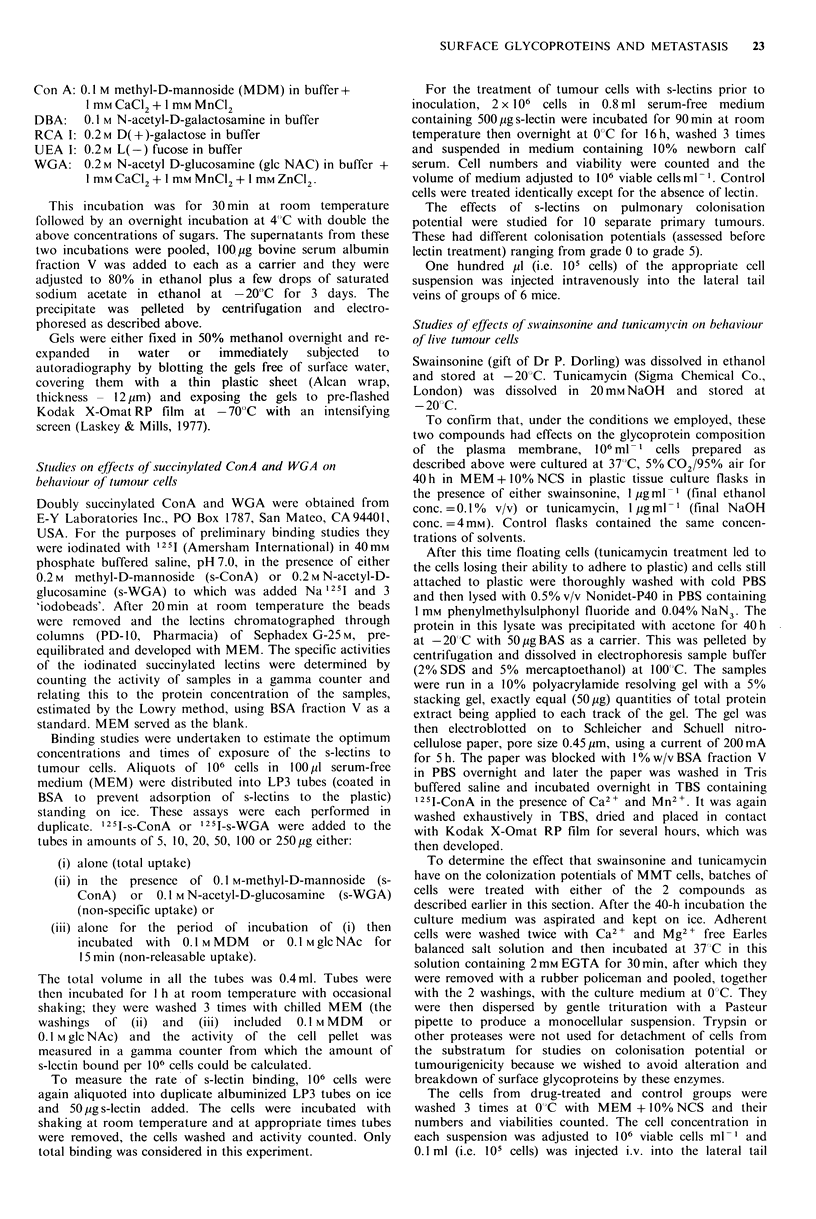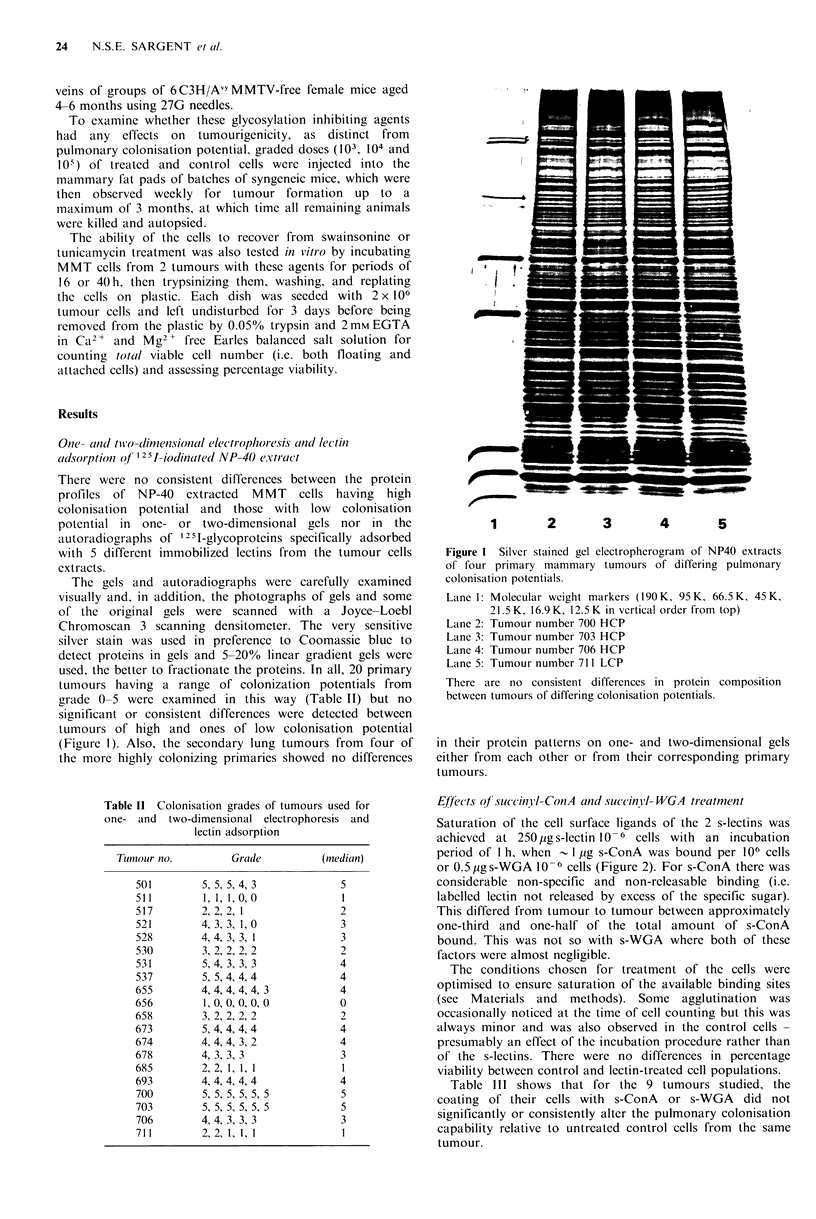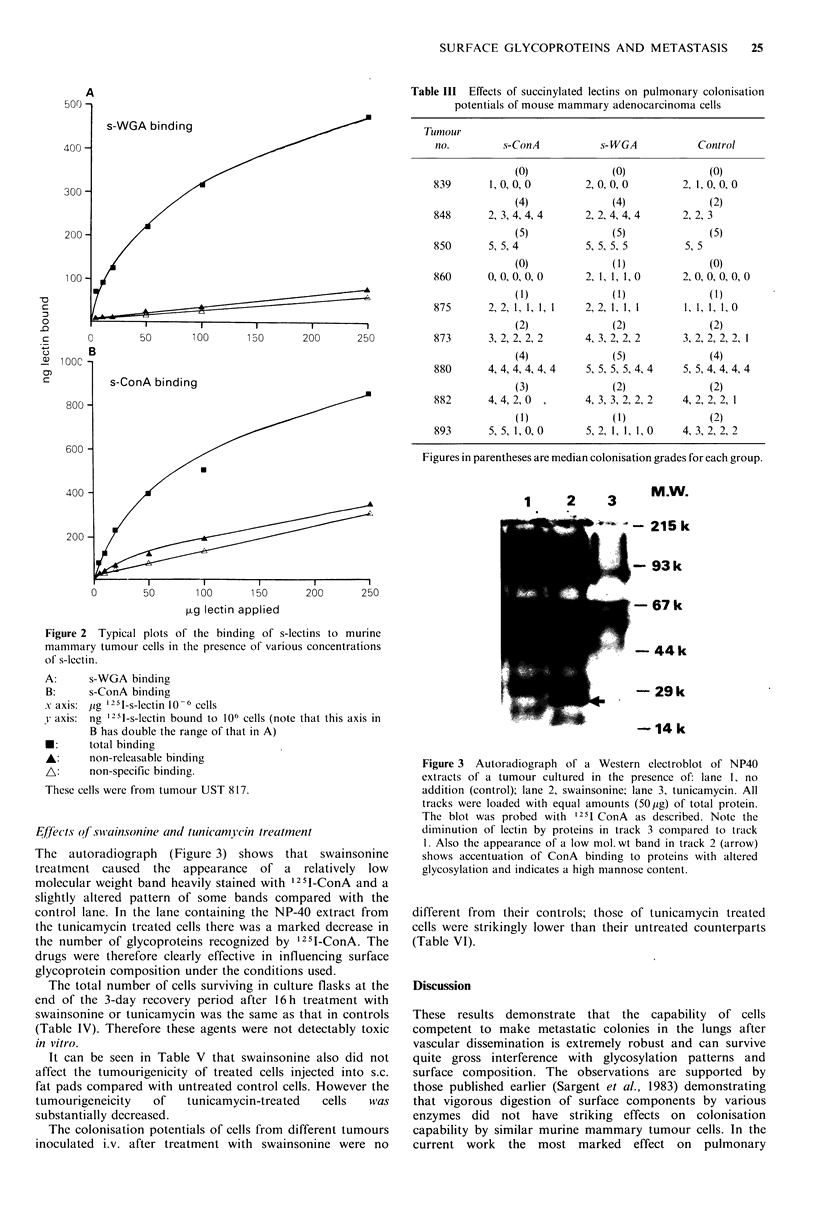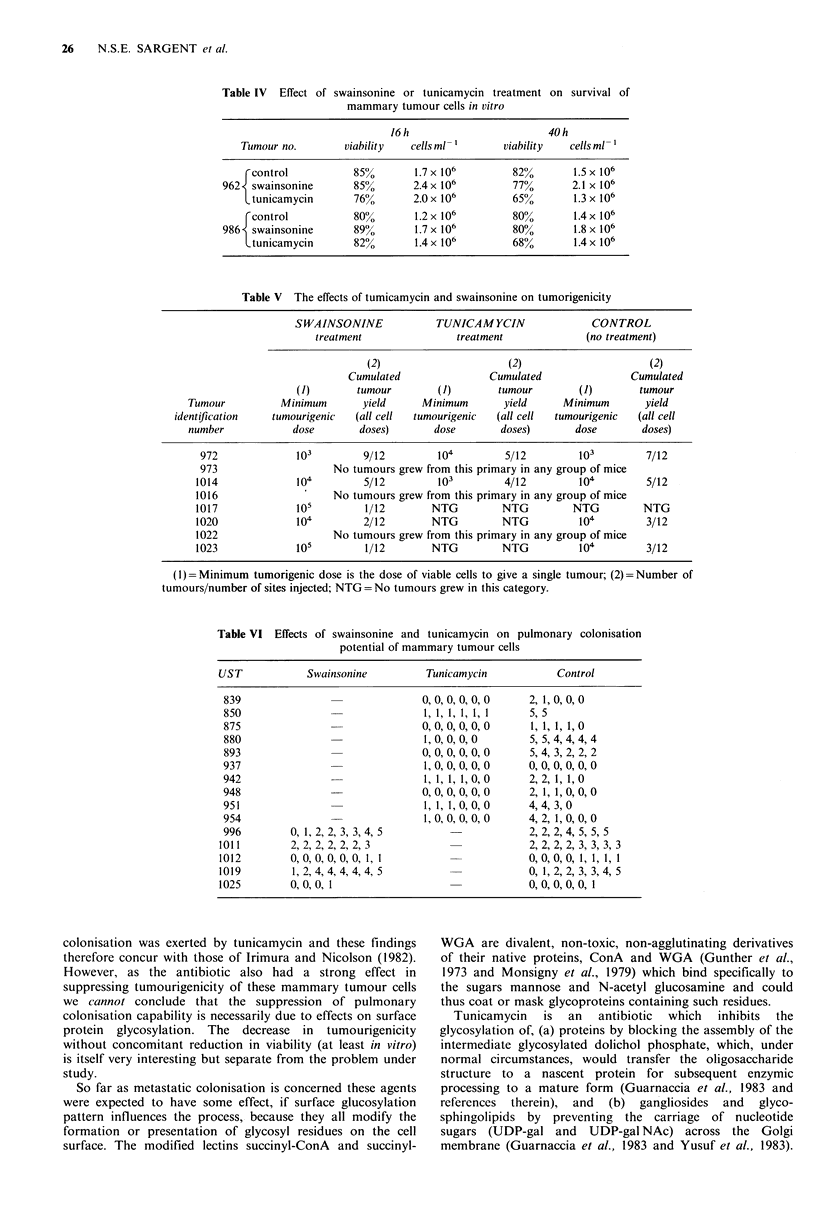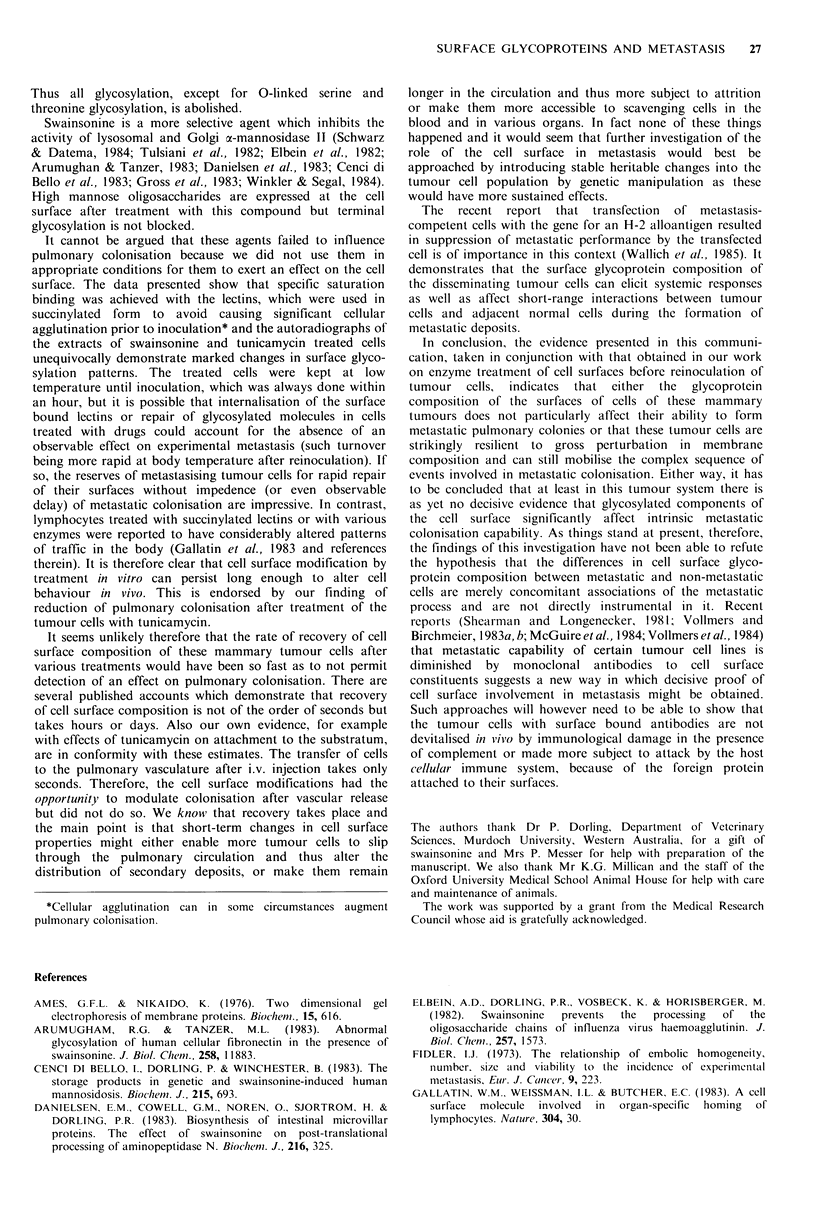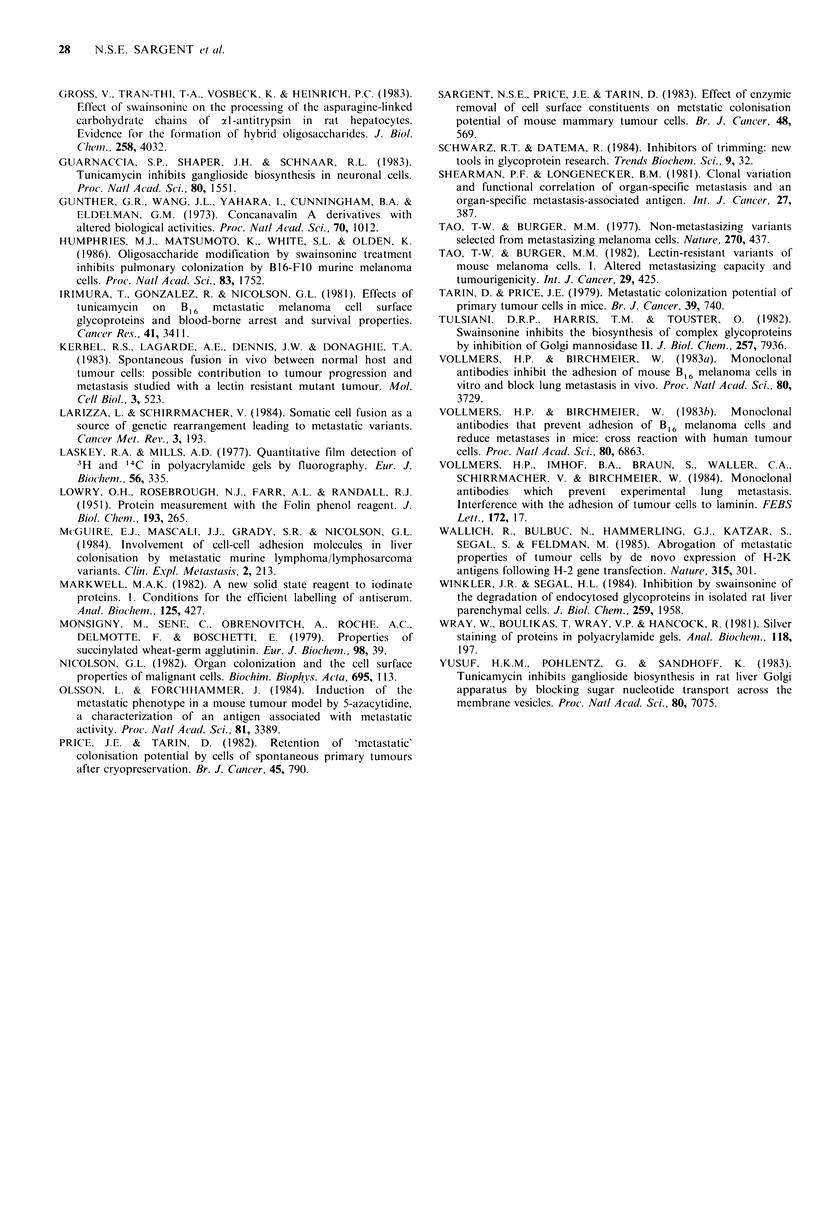# Effects of altering surface glycoprotein composition on metastatic colonisation potential of murine mammary tumour cells.

**DOI:** 10.1038/bjc.1987.5

**Published:** 1987-01

**Authors:** N. S. Sargent, J. E. Price, D. L. Darling, M. P. Flynn, D. Tarin

## Abstract

**Images:**


*Br. J. Cancer* (1987), **55**, 21–28 © The Macmillan Press Ltd., 1987

### Effects of altering surface glycoprotein composition on metastatic colonisation potential of murine mammary tumour cells

N.S.E. Sargent*, J.E. Price**, D.L. Darling, M.P. Flynn & D. Tarin

*Nuffield Department of Pathology, John Radcliffe Hospital, Oxford OX3 9DU, UK.*

**Summary** This study has examined cells from naturally-occurring murine mammary tumours to ascertain whether cell surface glycoproteins play a significant role in colonisation of the lungs after intravenous inoculation. It was found that gel electrophoretic analysis of membrane extracts and lectin adsorption studies did not reveal any consistent differences in glycoprotein composition of cells from tumours which can heavily colonise the lungs relative to ones from tumours which cannot do so or to cells from pulmonary metastases. Also, alteration of structural and functional properties of surface glycoproteins by treatment with succinylated lectins or with drugs such as tunicamycin and swainsonine, which inhibit glycosylation of membrane proteins, had no *specific* effects on metastatic colonisation of the lungs. Tunicamycin apparently decreased capability to form experimental metastases but also diminished tumourigenicity on subcutaneous inoculation, although it did not affect tumour cell viability *in vitro*.

This information supports earlier studies from this laboratory involving enzymic digestion of the surface of living tumour cells before inoculation and demonstrates that the pulmonary colonisation capability of these mammary tumour cells can withstand global disorganisation of membrane glycoprotein structure and composition. This implies that either the surface glycoproteins are not important in the colonisation process, or that these tumour cells have great capability for rapid repair of their surfaces. It is concluded that a clear answer to whether surface glycoprotein composition has a decisive role in pulmonary colonisation by these mammary tumour cells requires introduction of stable heritable traits into tumour cell populations by genetic manipulation.

The purpose of this investigation was to try to test directly whether glycosylated constituents of the cell surface play a significant part in metastatic colonisation of distant sites by disseminating tumour cells from naturally-occurring† (i.e. not transplanted) murine mammary tumours. Numerous publications have reported associations between the glycoprotein composition of the cell surface and metastatic behaviour (see Nicolson, 1982 for review) but very few have explored whether direct perturbation of the glycosylation patterns of surface molecules could demonstrate causal involvement in success or failure of the metastatic process. The most cogent evidence that the surface glycoprotein composition of tumour cells affects their metastatic capability has been provided by the work of Irimura *et al.* (1981), Kerbel *et al.* (1983), Larizza and Schirrmacher (1984), Olsson and Forchhammer (1984) and Humphries *et al.* (1986). All these groups observed alterations in capability to make metastatic tumour colonies in distant organs following procedures which concomitantly altered surface glycoprotein composition. Irimura *et al.* (1981) used tunicamycin, an antibiotic which blocks glycosylation of proteins and lipids and Humphries *et al.* (1986) used swainsonine which also affects glycosylation but at a different stage. Olsson and Forchhammer (1984) used 5-azacytidine which alters DNA methylation and hence gene expression and showed the appearance of a novel cell surface protein, whilst Kerbel *et al.* (1983) and Tao and Burger (1977, 1982) obtained cells with altered metastatic capability using *in vitro* selection for resistance to lectin toxicity and Larizza and Schirrmacher (1984) fused non-metastatic lymphoma cells with macrophages. The issue remains that none of these experimental procedures can be confidently assumed to have specific effects on the metastatic process *solely* via effects on the surface glycoprotein composition. Corroboration of this attractive hypothesis therefore requires converging lines of evidence from several experiments of different design on diverse tumour types.

The experiments described in this communication were performed to test whether direct perturbation of cell surface composition of cells from naturally-occurring murine mammary tumour would alter or interfere with their capability to colonise the lung which is their preferred site of metastasis.

### Materials and methods

### *Animals and tumours*

Primary and metastatic tumours were obtained from C3H/A$^{vy}$ mice endemically infected with the murine mammary tumour virus. Assays for metastatic potential were conducted in virus-free syngeneic animals of the same age and sex (see Tarin & Price, 1979, for details).

### *Cells and metastatic colonization assays*

Cells were aseptically obtained from primary murine mammary tumours growing in mice of the C3H/A$^{vy}$ strain. The tumours were finely minced with scalpels and incubated with collagenase, $1\,mg\,ml^{-1}$, in modified Eagle's medium (MEM) at 37°C for 2 h with constant agitation. Cells were kept free from any exogenous protein (e.g. bovine serum) throughout their preparation. At the end of this time, tissue clumps were allowed to settle under unit gravity and the supernatant collected (Tarin & Price, 1979). Suspended cells were washed once in MEM, resuspended in MEM and layered over isotonic Nycodenz [Nyegaard and Co., Oslo], diluted with MEM to give a density of $1.09\,g\,ml^{-1}$. The interface was gently stirred to produce a short gradient and this was centrifuged for 20 min at 1800 *g*. Cells at the

*Present address: Abteilung Biochemie, Biozentrum der Universitat Basel, Klingelbergstrasse 70, CH-4056 Basel, Switzerland.

**Present address: Department of Cell Biology (HMB-173), The University of Texas System Cancer Center, M.D. Anderson Hospital and Tumor Institute, Texas Medical Center, 6273 Bertner Avenue, Houston, Texas 77030, USA.

Correspondence: D. Tarin.
Received 6 May 1986; and in revised form, 15 September 1986.

†These tumours are caused by the murine mammary tumour virus (MMTV) and are referred to as naturally-occurring in this communication because they arise without the intervention of the investigator.

interface were collected, the procedure repeated and the tumour cells (now purified from erythrocytes and cell debris) were washed and suspended in MEM and kept on ice. An aliquot was stained with ethidium bromide and fluorescein diacetate and counted in a haemocytometer to assess total cell count and percentage viability (Price & Tarin, 1982).

When cells from pulmonary secondary deposits arising from i.v. inoculation of primary mammary tumour cells were studied, tumour nodules were carefully dissected free from lung tissue dissociated with collagenase and treated exactly as above.

The primary mammary tumour cells were assayed for their pulmonary metastatic colonization potential by i.v. inoculation of $5 \times 10^5$ viable cells in MEM into groups of 5–6 syngeneic MMTV-free female C3H/$A^{vy}$ mice under direct vision, following surgical exposure of one lateral tail vein of the mouse under methoxyflurane (Penthrane) anaesthesia. The mice were killed and autopsied 3 months later, or sooner if moribund, and the degree of pulmonary colonisation graded on a semi-quantitative scale (Table I) as previously described by Tarin and Price (1979). Lungs showing no surface evidence of tumour colonization were examined histopathologically to look for deeper deposits. Extrapulmonary lesions and any pulmonary foci not having the characteristic appearance of secondary mammary tumour deposits were also processed for histological examination.

**Table I** Scale for grading the colonisation potential of primary MMTVs

| *Number of secondary colonies seen on the surface of the lungs* | *Grade* | |
|---|---|---|
| 0 | 0 | Low colonisation potential |
| 1–9 | 1 | |
| 10–29 | 2 | |
| 30–49 | 3 | High colonisation potential |
| 50–99 | 4 | |
| >100 | 5 | |

*Extraction of cell membranes with Nonidet-P40*

Disaggregated tumour cells were centrifuged at 30*g* for 15 min, and the pellets resuspended in ice-cold 0.15 M NaCl, 10 mM EDTA, 20 mM Tris, pH 7.4. The cells were then immediately re-pelleted by centrifugation and resuspended in fresh buffer. This suspension was then adjusted to (final concentrations) 0.5% Nonidet-P40, 1 mM PMSF and 0.04% $NaN_3$; approximately 200 µl 0.5% NP40/$10^7$ cells. This was vortexed vigorously at room temperature for ~10 sec at 0, 5, 10 and 15 min to disrupt cells and solubilise their membranes and then centrifuged for 5 min in a Beckman microfuge B. The supernatants were stored in aliquots in liquid nitrogen with one aliquot being kept at −20°C (for subsequent Lowry protein estimation). The nuclear pellets were fixed in buffered isotonic formaldehyde and processed for histopathological examination to confirm effective solubilisation of cell membranes.

*Protein estimation*

The protein contents of solutions were determined by the method of Lowry *et al.* (1951), using the above lysis buffer as blank, with bovine serum albumin fraction V as standard. Absorbance was measured at 715 nm in a Pye Unicam SP1800 spectrophotometer.

*One dimensional electrophoresis*

Resolving slab gels were made of a linear 5–20% gradient of polyacrylamide, pH 8.8 (Trizma pre-set pH crystals), using a Pharmacia gradient mixer, with a 4% acrylamide stacking gel, pH 6.8. The running buffer was Tris-glycine-SDS, pH 8.8.

Samples were prepared by boiling in 2% SDS and 5% β-mercaptoethanol for 3 min before being loaded onto the gel (50 µg protein per lane) and run through the stacking gel at 35 mA/gel then at 50 mA/gel through the resolving gel. Bromophenol blue was used as a tracking dye and was run just off the end of the gels which were then fixed in 50% methanol overnight before being stained with silver according to the method of Wray *et al.* (1981).

*Two dimensional electrophoresis*

Rod gels of 2.7 × 60 mm were made consisting of 9 M urea, 4% Nonidet-P40, 2% ampholyte, pH 3–10 (Pharmalyte) in a 4% polyacrylamide gel. The cathode was 0.1 M NaOH and the anode 0.1% $H_3PO_4$. Samples were boiled in SDS and β-mercaptoethanol for 3 min. SDS was then displaced with a 10× excess of Nonidet P-40 (Ames & Nikaido, 1976) and 25 µg protein in 75 µl was loaded per gel, overlayered with 20 µl dilute sample buffer and electrophoresed at 400 V for 17 h. Rod gels were then equilibrated in 20% β-mercaptoethanol, 4% SDS, pH 6.8 for 70 min at room temperature and stored at −20°C until electrophoresis in the second dimension. For this they were thawed and fused to the stacking gel of gels made exactly as described above, using hot 2% agarose with 0.1% SDS, pH 6.8 containing bromophenol blue as a tracking dye.

*Protein $^{125}$I-iodination, immobilized-lectin adsorption and autoradiography of Nonidet-P40 extract*

The NP-40 extracts described above were adjusted to 2 mg protein $ml^{-1}$ in 0.7 ml (i.e. 1.4 mg protein). This solution was made to 80% in ethanol with a few drops of saturated sodium acetate in ethanol added (to improve flocculation). This was mixed and kept at −20°C for ~50 h. The precipitate was pelleted by centrifugation at 1800*g* for 20 min, washed once with 3 ml ethanol, vacuum dried and solubilised in 0.5 ml 50 mM PBS containing 2% SDS, pH 7.0 at 65°C for 2 h. Proteins were iodinated in this buffer at room temperature for 20 min using 150 µCi Na $^{125}$I per tube and the solid state reagent Iodo-beads, 1 bead per tube for the primary tumour extracts, 4 beads/tube for lung secondary extracts (Markwell, 1982). The cells from metastases yielded less protein and were therefore incubated with more beads to increase specific activity of labelling and improve sensitivity of detection. (This is a chemical, not enzymic, iodination reagent consisting of N-chlorobenzenesulphonamide, sodium salt, covalently attached to nonporous polystyrene beads). The samples were then kept on ice until being chromatographed through disposable mini-columns of Sephadex G-25 M, equilibrated and developed in 10 mM Tris. buffered saline, pH 7.4 containing 0.5% v/v Nonidet-P40 and 0.04% $NaN_3$. Material emerging in the void volume was collected and kept on ice.

Agarose-bound lectins (Eli Lilly, Windlesham, Surrey) were prepared by repeated washing in this buffer with the modification that, after removal of the phosphate buffer in which most lectins were stored, agarose-Con A was equilibrated with buffer + 2 mM $CaCl_2$ + 2 mM $MnCl_2$ and agarose-WGA with buffer + 2 mM $CaCl_2$ + 2 mM $MnCl_2$ + 2 mM $ZnCl_2$.

All 5 lectins were distributed into tubes in aliquots of 200 µl and equal amounts of $^{125}$I-labelled protein extracts added to them again in 200 µl volumes. This was incubated at 4°C overnight and for 2 h at room temperature, with shaking. Bound material was then pelleted by centrifugation at 1800*g* for 5 min and this was washed with 3 × 2.5 ml ice cold buffer (with 1 mM $Ca^{2+}$, $Mn^{2+}$, $Zn^{2+}$ as above). The lectin gels were then resuspended in 0.5 ml buffer containing the appropriate sugar specifically bound by each lectin as follows, to release glycoproteins:

Con A: 0.1 M methyl-D-mannoside (MDM) in buffer + 1 mM $CaCl_2$ + 1 mM $MnCl_2$

DBA: 0.1 M N-acetyl-D-galactosamine in buffer

RCA I: 0.2 M D(+)-galactose in buffer

UEA I: 0.2 M L(−) fucose in buffer

WGA: 0.2 M N-acetyl D-glucosamine (glc NAC) in buffer + 1 mM $CaCl_2$ + 1 mM $MnCl_2$ + 1 mM $ZnCl_2$.

This incubation was for 30 min at room temperature followed by an overnight incubation at 4°C with double the above concentrations of sugars. The supernatants from these two incubations were pooled, 100 μg bovine serum albumin fraction V was added to each as a carrier and they were adjusted to 80% in ethanol plus a few drops of saturated sodium acetate in ethanol at −20°C for 3 days. The precipitate was pelleted by centrifugation and electrophoresed as described above.

Gels were either fixed in 50% methanol overnight and re-expanded in water or immediately subjected to autoradiography by blotting the gels free of surface water, covering them with a thin plastic sheet (Alcan wrap, thickness – 12 μm) and exposing the gels to pre-flashed Kodak X-Omat RP film at −70°C with an intensifying screen (Laskey & Mills, 1977).

*Studies on effects of succinylated ConA and WGA on behaviour of tumour cells*

Doubly succinylated ConA and WGA were obtained from E-Y Laboratories Inc., PO Box 1787, San Mateo, CA 94401, USA. For the purposes of preliminary binding studies they were iodinated with $^{125}I$ (Amersham International) in 40 mM phosphate buffered saline, pH 7.0, in the presence of either 0.2 M methyl-D-mannoside (s-ConA) or 0.2 M N-acetyl-D-glucosamine (s-WGA) to which was added $Na^{125}I$ and 3 'iodobeads'. After 20 min at room temperature the beads were removed and the lectins chromatographed through columns (PD-10, Pharmacia) of Sephadex G-25 M, pre-equilibrated and developed with MEM. The specific activities of the iodinated succinylated lectins were determined by counting the activity of samples in a gamma counter and relating this to the protein concentration of the samples, estimated by the Lowry method, using BSA fraction V as a standard. MEM served as the blank.

Binding studies were undertaken to estimate the optimum concentrations and times of exposure of the s-lectins to tumour cells. Aliquots of $10^6$ cells in 100 μl serum-free medium (MEM) were distributed into LP3 tubes (coated in BSA to prevent adsorption of s-lectins to the plastic) standing on ice. These assays were each performed in duplicate. $^{125}I$-s-ConA or $^{125}I$-s-WGA were added to the tubes in amounts of 5, 10, 20, 50, 100 or 250 μg either:

(i) alone (total uptake)
(ii) in the presence of 0.1 M-methyl-D-mannoside (s-ConA) or 0.1 M N-acetyl-D-glucosamine (s-WGA) (non-specific uptake) or
(iii) alone for the period of incubation of (i) then incubated with 0.1 M MDM or 0.1 M glcNAc for 15 min (non-releasable uptake).

The total volume in all the tubes was 0.4 ml. Tubes were then incubated for 1 h at room temperature with occasional shaking; they were washed 3 times with chilled MEM (the washings of (ii) and (iii) included 0.1 M MDM or 0.1 M glcNAc) and the activity of the cell pellet was measured in a gamma counter from which the amount of s-lectin bound per $10^6$ cells could be calculated.

To measure the rate of s-lectin binding, $10^6$ cells were again aliquoted into duplicate albuminized LP3 tubes on ice and 50 μg s-lectin added. The cells were incubated with shaking at room temperature and at appropriate times tubes were removed, the cells washed and activity counted. Only total binding was considered in this experiment.

For the treatment of tumour cells with s-lectins prior to inoculation, $2 \times 10^6$ cells in 0.8 ml serum-free medium containing 500 μg s-lectin were incubated for 90 min at room temperature then overnight at 0°C for 16 h, washed 3 times and suspended in medium containing 10% newborn calf serum. Cell numbers and viability were counted and the volume of medium adjusted to $10^6$ viable cells $ml^{-1}$. Control cells were treated identically except for the absence of lectin.

The effects of s-lectins on pulmonary colonisation potential were studied for 10 separate primary tumours. These had different colonisation potentials (assessed before lectin treatment) ranging from grade 0 to grade 5).

One hundred μl (i.e. $10^5$ cells) of the appropriate cell suspension was injected intravenously into the lateral tail veins of groups of 6 mice.

*Studies of effects of swainsonine and tunicamycin on behaviour of live tumour cells*

Swainsonine (gift of Dr P. Dorling) was dissolved in ethanol and stored at −20°C. Tunicamycin (Sigma Chemical Co., London) was dissolved in 20 mM NaOH and stored at −20°C.

To confirm that, under the conditions we employed, these two compounds had effects on the glycoprotein composition of the plasma membrane, $10^6$ $ml^{-1}$ cells prepared as described above were cultured at 37°C, 5% $CO_2$/95% air for 40 h in MEM + 10% NCS in plastic tissue culture flasks in the presence of either swainsonine, 1 μg $ml^{-1}$ (final ethanol conc. = 0.1% v/v) or tunicamycin, 1 μg $ml^{-1}$ (final NaOH conc. = 4 mM). Control flasks contained the same concentrations of solvents.

After this time floating cells (tunicamycin treatment led to the cells losing their ability to adhere to plastic) and cells still attached to plastic were thoroughly washed with cold PBS and then lysed with 0.5% v/v Nonidet-P40 in PBS containing 1 mM phenylmethylsulphonyl fluoride and 0.04% $NaN_3$. The protein in this lysate was precipitated with acetone for 40 h at −20°C with 50 μg BAS as a carrier. This was pelleted by centrifugation and dissolved in electrophoresis sample buffer (2% SDS and 5% mercaptoethanol) at 100°C. The samples were run in a 10% polyacrylamide resolving gel with a 5% stacking gel, exactly equal (50 μg) quantities of total protein extract being applied to each track of the gel. The gel was then electroblotted on to Schleicher and Schuell nitrocellulose paper, pore size 0.45 μm, using a current of 200 mA for 5 h. The paper was blocked with 1% w/v BSA fraction V in PBS overnight and later the paper was washed in Tris buffered saline and incubated overnight in TBS containing $^{125}I$-ConA in the presence of $Ca^{2+}$ and $Mn^{2+}$. It was again washed exhaustively in TBS, dried and placed in contact with Kodak X-Omat RP film for several hours, which was then developed.

To determine the effect that swainsonine and tunicamycin have on the colonization potentials of MMT cells, batches of cells were treated with either of the 2 compounds as described earlier in this section. After the 40-h incubation the culture medium was aspirated and kept on ice. Adherent cells were washed twice with $Ca^{2+}$ and $Mg^{2+}$ free Earles balanced salt solution and then incubated at 37°C in this solution containing 2 mM EGTA for 30 min, after which they were removed with a rubber policeman and pooled, together with the 2 washings, with the culture medium at 0°C. They were then dispersed by gentle trituration with a Pasteur pipette to produce a monocellular suspension. Trypsin or other proteases were not used for detachment of cells from the substratum for studies on colonisation potential or tumourigenicity because we wished to avoid alteration and breakdown of surface glycoproteins by these enzymes.

The cells from drug-treated and control groups were washed 3 times at 0°C with MEM + 10% NCS and their numbers and viabilities counted. The cell concentration in each suspension was adjusted to $10^6$ viable cells $ml^{-1}$ and 0.1 ml (i.e. $10^5$ cells) was injected i.v. into the lateral tail veins of groups of 6 C3H/$A^{vy}$ MMTV-free female mice aged 4–6 months using 27G needles.

To examine whether these glycosylation inhibiting agents had any effects on tumourigenicity, as distinct from pulmonary colonisation potential, graded doses ($10^3$, $10^4$ and $10^5$) of treated and control cells were injected into the mammary fat pads of batches of syngeneic mice, which were then observed weekly for tumour formation up to a maximum of 3 months, at which time all remaining animals were killed and autopsied.

The ability of the cells to recover from swainsonine or tunicamycin treatment was also tested *in vitro* by incubating MMT cells from 2 tumours with these agents for periods of 16 or 40 h, then trypsinizing them, washing, and replating the cells on plastic. Each dish was seeded with $2\times10^6$ tumour cells and left undisturbed for 3 days before being removed from the plastic by 0.05% trypsin and 2 mM EGTA in $Ca^{2+}$ and $Mg^{2+}$ free Earles balanced salt solution for counting *total* viable cell number (i.e. both floating and attached cells) and assessing percentage viability.

### Results

### *One- and two-dimensional electrophoresis and lectin adsorption of $^{125}$I-iodinated NP-40 extract*

There were no consistent differences between the protein profiles of NP-40 extracted MMT cells having high colonisation potential and those with low colonisation potential in one- or two-dimensional gels nor in the autoradiographs of $^{125}$I-glycoproteins specifically adsorbed with 5 different immobilized lectins from the tumour cells extracts.

The gels and autoradiographs were carefully examined visually and, in addition, the photographs of gels and some of the original gels were scanned with a Joyce–Loebl Chromoscan 3 scanning densitometer. The very sensitive silver stain was used in preference to Coomassie blue to detect proteins in gels and 5–20% linear gradient gels were used, the better to fractionate the proteins. In all, 20 primary tumours having a range of colonization potentials from grade 0–5 were examined in this way (Table II) but no significant or consistent differences were detected between tumours of high and ones of low colonisation potential (Figure 1). Also, the secondary lung tumours from four of the more highly colonizing primaries showed no differences in their protein patterns on one- and two-dimensional gels either from each other or from their corresponding primary tumours.

**Figure 1** Silver stained gel electropherogram of NP40 extracts of four primary mammary tumours of differing pulmonary colonisation potentials.

Lane 1: Molecular weight markers (190 K, 95 K, 66.5 K, 45 K, 21.5 K, 16.9 K, 12.5 K in vertical order from top)
Lane 2: Tumour number 700 HCP
Lane 3: Tumour number 703 HCP
Lane 4: Tumour number 706 HCP
Lane 5: Tumour number 711 LCP

There are no consistent differences in protein composition between tumours of differing colonisation potentials.

**Table II** Colonisation grades of tumours used for one- and two-dimensional electrophoresis and lectin adsorption

| *Tumour no.* | *Grade* | *(median)* |
|---|---|---|
| 501 | 5, 5, 5, 4, 3 | 5 |
| 511 | 1, 1, 1, 0, 0 | 1 |
| 517 | 2, 2, 2, 1 | 2 |
| 521 | 4, 3, 3, 1, 0 | 3 |
| 528 | 4, 4, 3, 3, 1 | 3 |
| 530 | 3, 2, 2, 2, 2 | 2 |
| 531 | 5, 4, 3, 3, 3 | 4 |
| 537 | 5, 5, 4, 4, 4 | 4 |
| 655 | 4, 4, 4, 4, 4, 3 | 4 |
| 656 | 1, 0, 0, 0, 0, 0 | 0 |
| 658 | 3, 2, 2, 2, 2 | 2 |
| 673 | 5, 4, 4, 4, 4 | 4 |
| 674 | 4, 4, 4, 3, 2 | 4 |
| 678 | 4, 3, 3, 3 | 3 |
| 685 | 2, 2, 1, 1, 1 | 1 |
| 693 | 4, 4, 4, 4, 4 | 4 |
| 700 | 5, 5, 5, 5, 5, 5 | 5 |
| 703 | 5, 5, 5, 5, 5, 5 | 5 |
| 706 | 4, 4, 3, 3, 3 | 3 |
| 711 | 2, 2, 1, 1, 1 | 1 |

### *Effects of succinyl-ConA and succinyl-WGA treatment*

Saturation of the cell surface ligands of the 2 s-lectins was achieved at 250 $\mu$g s-lectin $10^{-6}$ cells with an incubation period of 1 h, when $\sim$1 $\mu$g s-ConA was bound per $10^6$ cells or 0.5 $\mu$g s-WGA $10^{-6}$ cells (Figure 2). For s-ConA there was considerable non-specific and non-releasable binding (i.e. labelled lectin not released by excess of the specific sugar). This differed from tumour to tumour between approximately one-third and one-half of the total amount of s-ConA bound. This was not so with s-WGA where both of these factors were almost negligible.

The conditions chosen for treatment of the cells were optimised to ensure saturation of the available binding sites (see Materials and methods). Some agglutination was occasionally noticed at the time of cell counting but this was always minor and was also observed in the control cells – presumably an effect of the incubation procedure rather than of the s-lectins. There were no differences in percentage viability between control and lectin-treated cell populations.

Table III shows that for the 9 tumours studied, the coating of their cells with s-ConA or s-WGA did not significantly or consistently alter the pulmonary colonisation capability relative to untreated control cells from the same tumour.

**Figure 2** Typical plots of the binding of s-lectins to murine mammary tumour cells in the presence of various concentrations of s-lectin.

A: s-WGA binding

B: s-ConA binding

*x* axis: μg $^{125}$I-s-lectin $10^{-6}$ cells

*y* axis: ng $^{125}$I-s-lectin bound to $10^6$ cells (note that this axis in B has double the range of that in A)

■: total binding

▲: non-releasable binding

△: non-specific binding.

These cells were from tumour UST 817.

*Effects of swainsonine and tunicamycin treatment*

The autoradiograph (Figure 3) shows that swainsonine treatment caused the appearance of a relatively low molecular weight band heavily stained with $^{125}$I-ConA and a slightly altered pattern of some bands compared with the control lane. In the lane containing the NP-40 extract from the tunicamycin treated cells there was a marked decrease in the number of glycoproteins recognized by $^{125}$I-ConA. The drugs were therefore clearly effective in influencing surface glycoprotein composition under the conditions used.

The total number of cells surviving in culture flasks at the end of the 3-day recovery period after 16 h treatment with swainsonine or tunicamycin was the same as that in controls (Table IV). Therefore these agents were not detectably toxic *in vitro*.

It can be seen in Table V that swainsonine also did not affect the tumourigenicity of treated cells injected into s.c. fat pads compared with untreated control cells. However the tumourigeneicity of tunicamycin-treated cells *was* substantially decreased.

The colonisation potentials of cells from different tumours inoculated i.v. after treatment with swainsonine were no different from their controls; those of tunicamycin treated cells were strikingly lower than their untreated counterparts (Table VI).

**Table III** Effects of succinylated lectins on pulmonary colonisation potentials of mouse mammary adenocarcinoma cells

| *Tumour no.* | *s-ConA* | *s-WGA* | *Control* |
|---|---|---|---|
| 839 | (0) 1, 0, 0, 0 | (0) 2, 0, 0, 0 | (0) 2, 1, 0, 0, 0 |
| 848 | (4) 2, 3, 4, 4, 4 | (4) 2, 2, 4, 4, 4 | (2) 2, 2, 3 |
| 850 | (5) 5, 5, 4 | (5) 5, 5, 5, 5 | (5) 5, 5 |
| 860 | (0) 0, 0, 0, 0, 0 | (1) 2, 1, 1, 1, 0 | (0) 2, 0, 0, 0, 0, 0 |
| 875 | (1) 2, 2, 1, 1, 1, 1 | (1) 2, 2, 1, 1, 1 | (1) 1, 1, 1, 1, 0 |
| 873 | (2) 3, 2, 2, 2, 2 | (2) 4, 3, 2, 2, 2 | (2) 3, 2, 2, 2, 2, 1 |
| 880 | (4) 4, 4, 4, 4, 4, 4 | (5) 5, 5, 5, 5, 4, 4 | (4) 5, 5, 4, 4, 4, 4 |
| 882 | (3) 4, 4, 2, 0 , | (2) 4, 3, 3, 2, 2, 2 | (2) 4, 2, 2, 2, 1 |
| 893 | (1) 5, 5, 1, 0, 0 | (1) 5, 2, 1, 1, 1, 0 | (2) 4, 3, 2, 2, 2 |

Figures in parentheses are median colonisation grades for each group.

**Figure 3** Autoradiograph of a Western electroblot of NP40 extracts of a tumour cultured in the presence of: lane 1, no addition (control); lane 2, swainsonine; lane 3, tunicamycin. All tracks were loaded with equal amounts (50 μg) of total protein. The blot was probed with $^{125}$I ConA as described. Note the diminution of lectin by proteins in track 3 compared to track 1. Also the appearance of a low mol. wt band in track 2 (arrow) shows accentuation of ConA binding to proteins with altered glycosylation and indicates a high mannose content.

### Discussion

These results demonstrate that the capability of cells competent to make metastatic colonies in the lungs after vascular dissemination is extremely robust and can survive quite gross interference with glycosylation patterns and surface composition. The observations are supported by those published earlier (Sargent *et al.*, 1983) demonstrating that vigorous digestion of surface components by various enzymes did not have striking effects on colonisation capability by similar murine mammary tumour cells. In the current work the most marked effect on pulmonary

**Table IV** Effect of swainsonine or tunicamycin treatment on survival of mammary tumour cells *in vitro*

| *Tumour no.* | | *16 h* viability | *16 h* cells ml$^{-1}$ | *40 h* viability | *40 h* cells ml$^{-1}$ |
|---|---|---|---|---|---|
| 962 | control | 85% | $1.7 \times 10^6$ | 82% | $1.5 \times 10^6$ |
| | swainsonine | 85% | $2.4 \times 10^6$ | 77% | $2.1 \times 10^6$ |
| | tunicamycin | 76% | $2.0 \times 10^6$ | 65% | $1.3 \times 10^6$ |
| 986 | control | 80% | $1.2 \times 10^6$ | 80% | $1.4 \times 10^6$ |
| | swainsonine | 89% | $1.7 \times 10^6$ | 80% | $1.8 \times 10^6$ |
| | tunicamycin | 82% | $1.4 \times 10^6$ | 68% | $1.4 \times 10^6$ |

**Table V** The effects of tumicamycin and swainsonine on tumorigenicity

| *Tumour identification number* | *SWAINSONINE treatment* (*1*) *Minimum tumourigenic dose* | *SWAINSONINE treatment* (*2*) *Cumulated tumour yield* (*all cell doses*) | *TUNICAMYCIN treatment* (*1*) *Minimum tumourigenic dose* | *TUNICAMYCIN treatment* (*2*) *Cumulated tumour yield* (*all cell doses*) | *CONTROL* (*no treatment*) (*1*) *Minimum tumourigenic dose* | *CONTROL* (*no treatment*) (*2*) *Cumulated tumour yield* (*all cell doses*) |
|---|---|---|---|---|---|---|
| 972 | $10^3$ | 9/12 | $10^4$ | 5/12 | $10^3$ | 7/12 |
| 973 | | No tumours grew from this primary in any group of mice | | | | |
| 1014 | $10^4$ | 5/12 | $10^3$ | 4/12 | $10^4$ | 5/12 |
| 1016 | | No tumours grew from this primary in any group of mice | | | | |
| 1017 | $10^5$ | 1/12 | NTG | NTG | NTG | NTG |
| 1020 | $10^4$ | 2/12 | NTG | NTG | $10^4$ | 3/12 |
| 1022 | | No tumours grew from this primary in any group of mice | | | | |
| 1023 | $10^5$ | 1/12 | NTG | NTG | $10^4$ | 3/12 |

(1) = Minimum tumorigenic dose is the dose of viable cells to give a single tumour; (2) = Number of tumours/number of sites injected; NTG = No tumours grew in this category.

**Table VI** Effects of swainsonine and tunicamycin on pulmonary colonisation potential of mammary tumour cells

| *UST* | *Swainsonine* | *Tunicamycin* | *Control* |
|---|---|---|---|
| 839 | — | 0, 0, 0, 0, 0, 0 | 2, 1, 0, 0, 0 |
| 850 | — | 1, 1, 1, 1, 1, 1 | 5, 5 |
| 875 | — | 0, 0, 0, 0, 0, 0 | 1, 1, 1, 1, 0 |
| 880 | — | 1, 0, 0, 0, 0 | 5, 5, 4, 4, 4, 4 |
| 893 | — | 0, 0, 0, 0, 0, 0 | 5, 4, 3, 2, 2, 2 |
| 937 | — | 1, 0, 0, 0, 0, 0 | 0, 0, 0, 0, 0, 0 |
| 942 | — | 1, 1, 1, 1, 0, 0 | 2, 2, 1, 1, 0 |
| 948 | — | 0, 0, 0, 0, 0, 0 | 2, 1, 1, 0, 0, 0 |
| 951 | — | 1, 1, 1, 0, 0, 0 | 4, 4, 3, 0 |
| 954 | — | 1, 0, 0, 0, 0, 0 | 4, 2, 1, 0, 0, 0 |
| 996 | 0, 1, 2, 2, 3, 3, 4, 5 | — | 2, 2, 2, 4, 5, 5, 5 |
| 1011 | 2, 2, 2, 2, 2, 2, 3 | — | 2, 2, 2, 2, 3, 3, 3, 3 |
| 1012 | 0, 0, 0, 0, 0, 0, 1, 1 | — | 0, 0, 0, 0, 1, 1, 1, 1 |
| 1019 | 1, 2, 4, 4, 4, 4, 4, 5 | — | 0, 1, 2, 2, 3, 3, 4, 5 |
| 1025 | 0, 0, 0, 1 | — | 0, 0, 0, 0, 0, 1 |

colonisation was exerted by tunicamycin and these findings therefore concur with those of Irimura and Nicolson (1982). However, as the antibiotic also had a strong effect in suppressing tumourigenicity of these mammary tumour cells we *cannot* conclude that the suppression of pulmonary colonisation capability is necessarily due to effects on surface protein glycosylation. The decrease in tumourigenicity without concomitant reduction in viability (at least *in vitro*) is itself very interesting but separate from the problem under study.

So far as metastatic colonisation is concerned these agents were expected to have some effect, if surface glucosylation pattern influences the process, because they all modify the formation or presentation of glycosyl residues on the cell surface. The modified lectins succinyl-ConA and succinyl-WGA are divalent, non-toxic, non-agglutinating derivatives of their native proteins, ConA and WGA (Gunther *et al.*, 1973 and Monsigny *et al.*, 1979) which bind specifically to the sugars mannose and N-acetyl glucosamine and could thus coat or mask glycoproteins containing such residues.

Tunicamycin is an antibiotic which inhibits the glycosylation of, (a) proteins by blocking the assembly of the intermediate glycosylated dolichol phosphate, which, under normal circumstances, would transfer the oligosaccharide structure to a nascent protein for subsequent enzymic processing to a mature form (Guarnaccia *et al.*, 1983 and references therein), and (b) gangliosides and glycosphingolipids by preventing the carriage of nucleotide sugars (UDP-gal and UDP-gal NAc) across the Golgi membrane (Guarnaccia *et al.*, 1983 and Yusuf *et al.*, 1983).

Thus all glycosylation, except for O-linked serine and threonine glycosylation, is abolished.

Swainsonine is a more selective agent which inhibits the activity of lysosomal and Golgi α-mannosidase II (Schwarz & Datema, 1984; Tulsiani *et al.*, 1982; Elbein *et al.*, 1982; Arumughan & Tanzer, 1983; Danielsen *et al.*, 1983; Cenci di Bello *et al.*, 1983; Gross *et al.*, 1983; Winkler & Segal, 1984). High mannose oligosaccharides are expressed at the cell surface after treatment with this compound but terminal glycosylation is not blocked.

It cannot be argued that these agents failed to influence pulmonary colonisation because we did not use them in appropriate conditions for them to exert an effect on the cell surface. The data presented show that specific saturation binding was achieved with the lectins, which were used in succinylated form to avoid causing significant cellular agglutination prior to inoculation* and the autoradiographs of the extracts of swainsonine and tunicamycin treated cells unequivocally demonstrate marked changes in surface glycosylation patterns. The treated cells were kept at low temperature until inoculation, which was always done within an hour, but it is possible that internalisation of the surface bound lectins or repair of glycosylated molecules in cells treated with drugs could account for the absence of an observable effect on experimental metastasis (such turnover being more rapid at body temperature after reinoculation). If so, the reserves of metastasising tumour cells for rapid repair of their surfaces without impedence (or even observable delay) of metastatic colonisation are impressive. In contrast, lymphocytes treated with succinylated lectins or with various enzymes were reported to have considerably altered patterns of traffic in the body (Gallatin *et al.*, 1983 and references therein). It is therefore clear that cell surface modification by treatment *in vitro* can persist long enough to alter cell behaviour *in vivo*. This is endorsed by our finding of reduction of pulmonary colonisation after treatment of the tumour cells with tunicamycin.

It seems unlikely therefore that the rate of recovery of cell surface composition of these mammary tumour cells after various treatments would have been so fast as to not permit detection of an effect on pulmonary colonisation. There are several published accounts which demonstrate that recovery of cell surface composition is not of the order of seconds but takes hours or days. Also our own evidence, for example with effects of tunicamycin on attachment to the substratum, are in conformity with these estimates. The transfer of cells to the pulmonary vasculature after i.v. injection takes only seconds. Therefore, the cell surface modifications had the *opportunity* to modulate colonisation after vascular release but did not do so. We *know* that recovery takes place and the main point is that short-term changes in cell surface properties might either enable more tumour cells to slip through the pulmonary circulation and thus alter the distribution of secondary deposits, or make them remain longer in the circulation and thus more subject to attrition or make them more accessible to scavenging cells in the blood and in various organs. In fact none of these things happened and it would seem that further investigation of the role of the cell surface in metastasis would best be approached by introducing stable heritable changes into the tumour cell population by genetic manipulation as these would have more sustained effects.

The recent report that transfection of metastasis-competent cells with the gene for an H-2 alloantigen resulted in suppression of metastatic performance by the transfected cell is of importance in this context (Wallich *et al.*, 1985). It demonstrates that the surface glycoprotein composition of the disseminating tumour cells can elicit systemic responses as well as affect short-range interactions between tumour cells and adjacent normal cells during the formation of metastatic deposits.

In conclusion, the evidence presented in this communication, taken in conjunction with that obtained in our work on enzyme treatment of cell surfaces before reinoculation of tumour cells, indicates that either the glycoprotein composition of the surfaces of cells of these mammary tumours does not particularly affect their ability to form metastatic pulmonary colonies or that these tumour cells are strikingly resilient to gross perturbation in membrane composition and can still mobilise the complex sequence of events involved in metastatic colonisation. Either way, it has to be concluded that at least in this tumour system there is as yet no decisive evidence that glycosylated components of the cell surface significantly affect intrinsic metastatic colonisation capability. As things stand at present, therefore, the findings of this investigation have not been able to refute the hypothesis that the differences in cell surface glycoprotein composition between metastatic and non-metastatic cells are merely concomitant associations of the metastatic process and are not directly instrumental in it. Recent reports (Shearman and Longenecker, 1981; Vollmers and Birchmeier, 1983*a*, *b*; McGuire *et al.*, 1984; Vollmers *et al.*, 1984) that metastatic capability of certain tumour cell lines is diminished by monoclonal antibodies to cell surface constituents suggests a new way in which decisive proof of cell surface involvement in metastasis might be obtained. Such approaches will however need to be able to show that the tumour cells with surface bound antibodies are not devitalised *in vivo* by immunological damage in the presence of complement or made more subject to attack by the host *cellular* immune system, because of the foreign protein attached to their surfaces.

*Cellular agglutination can in some circumstances augment pulmonary colonisation.

The authors thank Dr P. Dorling, Department of Veterinary Sciences, Murdoch University, Western Australia, for a gift of swainsonine and Mrs P. Messer for help with preparation of the manuscript. We also thank Mr K.G. Millican and the staff of the Oxford University Medical School Animal House for help with care and maintenance of animals.

The work was supported by a grant from the Medical Research Council whose aid is gratefully acknowledged.

**References**

AMES, G.F.L. & NIKAIDO, K. (1976). Two dimensional gel electrophoresis of membrane proteins. *Biochem.*, **15**, 616.

ARUMUGHAM, R.G. & TANZER, M.L. (1983). Abnormal glycosylation of human cellular fibronectin in the presence of swainsonine. *J. Biol. Chem.*, **258**, 11883.

CENCI DI BELLO, I., DORLING, P. & WINCHESTER, B. (1983). The storage products in genetic and swainsonine-induced human mannosidosis. *Biochem. J.*, **215**, 693.

DANIELSEN, E.M., COWELL, G.M., NOREN, O., SJORTROM, H. & DORLING, P.R. (1983). Biosynthesis of intestinal microvillar proteins. The effect of swainsonine on post-translational processing of aminopeptidase N. *Biochem. J.*, **216**, 325.

ELBEIN, A.D., DORLING, P.R., VOSBECK, K. & HORISBERGER, M. (1982). Swainsonine prevents the processing of the oligosaccharide chains of influenza virus haemoagglutinin. *J. Biol. Chem.*, **257**, 1573.

FIDLER, I.J. (1973). The relationship of embolic homogeneity, number, size and viability to the incidence of experimental metastasis. *Eur. J. Cancer*, **9**, 223.

GALLATIN, W.M., WEISSMAN, I.L. & BUTCHER, E.C. (1983). A cell surface molecule involved in organ-specific homing of lymphocytes. *Nature*, **304**, 30.

GROSS, V., TRAN-THI, T-A., VOSBECK, K. & HEINRICH, P.C. (1983). Effect of swainsonine on the processing of the asparagine-linked carbohydrate chains of α1-antitrypsin in rat hepatocytes. Evidence for the formation of hybrid oligosaccharides. *J. Biol. Chem.*, **258**, 4032.

GUARNACCIA, S.P., SHAPER, J.H. & SCHNAAR, R.L. (1983). Tunicamycin inhibits ganglioside biosynthesis in neuronal cells. *Proc. Natl Acad. Sci.*, **80**, 1551.

GUNTHER, G.R., WANG, J.L., YAHARA, I., CUNNINGHAM, B.A. & ELDELMAN, G.M. (1973). Concanavalin A derivatives with altered biological activities. *Proc. Natl Acad. Sci.*, **70**, 1012.

HUMPHRIES, M.J., MATSUMOTO, K., WHITE, S.L. & OLDEN, K. (1986). Oligosaccharide modification by swainsonine treatment inhibits pulmonary colonization by B16-F10 murine melanoma cells. *Proc. Natl Acad. Sci.*, **83**, 1752.

IRIMURA, T., GONZALEZ, R. & NICOLSON, G.L. (1981). Effects of tunicamycin on $B_{16}$ metastatic melanoma cell surface glycoproteins and blood-borne arrest and survival properties. *Cancer Res.*, **41**, 3411.

KERBEL, R.S., LAGARDE, A.E., DENNIS, J.W. & DONAGHIE, T.A. (1983). Spontaneous fusion in vivo between normal host and tumour cells: possible contribution to tumour progression and metastasis studied with a lectin resistant mutant tumour. *Mol. Cell Biol.*, **3**, 523.

LARIZZA, L. & SCHIRRMACHER, V. (1984). Somatic cell fusion as a source of genetic rearrangement leading to metastatic variants. *Cancer Met. Rev.*, **3**, 193.

LASKEY, R.A. & MILLS, A.D. (1977). Quantitative film detection of $^{3}H$ and $^{14}C$ in polyacrylamide gels by fluorography. *Eur. J. Biochem.*, **56**, 335.

LOWRY, O.H., ROSEBROUGH, N.J., FARR, A.L. & RANDALL, R.J. (1951). Protein measurement with the Folin phenol reagent. *J. Biol. Chem.*, **193**, 265.

McGUIRE, E.J., MASCALI, J.J., GRADY, S.R. & NICOLSON, G.L. (1984). Involvement of cell-cell adhesion molecules in liver colonisation by metastatic murine lymphoma/lymphosarcoma variants. *Clin. Expl. Metastasis*, **2**, 213.

MARKWELL, M.A.K. (1982). A new solid state reagent to iodinate proteins. I. Conditions for the efficient labelling of antiserum. *Anal. Biochem.*, **125**, 427.

MONSIGNY, M., SENE, C., OBRENOVITCH, A., ROCHE, A.C., DELMOTTE, F. & BOSCHETTI, E. (1979). Properties of succinylated wheat-germ agglutinin. *Eur. J. Biochem.*, **98**, 39.

NICOLSON, G.L. (1982). Organ colonization and the cell surface properties of malignant cells. *Biochim. Biophys. Acta*, **695**, 113.

OLSSON, L. & FORCHHAMMER, J. (1984). Induction of the metastatic phenotype in a mouse tumour model by 5-azacytidine, a characterization of an antigen associated with metastatic activity. *Proc. Natl Acad. Sci.*, **81**, 3389.

PRICE, J.E. & TARIN, D. (1982). Retention of 'metastatic' colonisation potential by cells of spontaneous primary tumours after cryopreservation. *Br. J. Cancer*, **45**, 790.

SARGENT, N.S.E., PRICE, J.E. & TARIN, D. (1983). Effect of enzymic removal of cell surface constituents on metstatic colonisation potential of mouse mammary tumour cells. *Br. J. Cancer*, **48**, 569.

SCHWARZ, R.T. & DATEMA, R. (1984). Inhibitors of trimming: new tools in glycoprotein research. *Trends Biochem. Sci.*, **9**, 32.

SHEARMAN, P.F. & LONGENECKER, B.M. (1981). Clonal variation and functional correlation of organ-specific metastasis and an organ-specific metastasis-associated antigen. *Int. J. Cancer*, **27**, 387.

TAO, T-W. & BURGER, M.M. (1977). Non-metastasizing variants selected from metastasizing melanoma cells. *Nature*, **270**, 437.

TAO, T-W. & BURGER, M.M. (1982). Lectin-resistant variants of mouse melanoma cells. I. Altered metastasizing capacity and tumourigenicity. *Int. J. Cancer*, **29**, 425.

TARIN, D. & PRICE, J.E. (1979). Metastatic colonization potential of primary tumour cells in mice. *Br. J. Cancer*, **39**, 740.

TULSIANI, D.R.P., HARRIS, T.M. & TOUSTER, O. (1982). Swainsonine inhibits the biosynthesis of complex glycoproteins by inhibition of Golgi mannosidase II. *J. Biol. Chem.*, **257**, 7936.

VOLLMERS, H.P. & BIRCHMEIER, W. (1983*a*). Monoclonal antibodies inhibit the adhesion of mouse $B_{16}$ melanoma cells in vitro and block lung metastasis in vivo. *Proc. Natl Acad. Sci.*, **80**, 3729.

VOLLMERS, H.P. & BIRCHMEIER, W. (1983*b*). Monoclonal antibodies that prevent adhesion of $B_{16}$ melanoma cells and reduce metastases in mice: cross reaction with human tumour cells. *Proc. Natl Acad. Sci.*, **80**, 6863.

VOLLMERS, H.P., IMHOF, B.A., BRAUN, S., WALLER, C.A., SCHIRRMACHER, V. & BIRCHMEIER, W. (1984). Monoclonal antibodies which prevent experimental lung metastasis. Interference with the adhesion of tumour cells to laminin. *FEBS Lett.*, **172**, 17.

WALLICH, R., BULBUC, N., HAMMERLING, G.J., KATZAR, S., SEGAL, S. & FELDMAN, M. (1985). Abrogation of metastatic properties of tumour cells by de novo expression of H-2K antigens following H-2 gene transfection. *Nature*, **315**, 301.

WINKLER, J.R. & SEGAL, H.L. (1984). Inhibition by swainsonine of the degradation of endocytosed glycoproteins in isolated rat liver parenchymal cells. *J. Biol. Chem.*, **259**, 1958.

WRAY, W., BOULIKAS, T. WRAY, V.P. & HANCOCK, R. (1981). Silver staining of proteins in polyacrylamide gels. *Anal. Biochem.*, **118**, 197.

YUSUF, H.K.M., POHLENTZ, G. & SANDHOFF, K. (1983). Tunicamycin inhibits ganglioside biosynthesis in rat liver Golgi apparatus by blocking sugar nucleotide transport across the membrane vesicles. *Proc. Natl Acad. Sci.*, **80**, 7075.